# Long term adjuvant endocrine therapy and risk of cardiovascular disease in female breast cancer survivors: systematic review

**DOI:** 10.1136/bmj.k3845

**Published:** 2018-10-08

**Authors:** Anthony Matthews, Susannah Stanway, Ruth E Farmer, Helen Strongman, Sara Thomas, Alexander R Lyon, Liam Smeeth, Krishnan Bhaskaran

**Affiliations:** 1 Department of Non-Communicable Diseases Epidemiology, London School of Hygiene and Tropical Medicine, London, UK; 2 Royal Marsden Hospital, London, UK; 3 Faculty of Medicine, Imperial College London, London, UK; 4 Royal Brompton Hospital, London, UK

## Abstract

**Objective:**

To investigate the effect of endocrine therapies on a wide range of specific clinical cardiovascular disease outcomes in women with a history of non-metastatic breast cancer.

**Design:**

Systematic review and meta-analysis of randomised controlled trials and observational studies.

**Data sources:**

Medline and Embase up until June 2018.

**Eligibility criteria for selecting studies:**

Studies were included if they investigated the risk of a specific cardiovascular disease outcome associated with use of either tamoxifen or an aromatase inhibitor, or compared the two treatments, in women with a history of non-metastatic breast cancer.

**Appraisal and data extraction:**

Relevant studies were originally identified and results extracted by one researcher, with a full replication of the study identification process by a combination of two other researchers. The Cochrane Collaboration’s tool for assessing risk of bias was used to assess risk of bias in randomised controlled trials, and this tool was adapted to assess risk of bias in observational studies.

**Results:**

26 studies were identified, with results for seven specific cardiovascular disease outcomes (venous thromboembolism, myocardial infarction, stroke, angina, heart failure, arrhythmia, and peripheral vascular disease). Results suggested an increased risk of venous thromboembolism in tamoxifen users compared with both non-users and aromatase inhibitor users. Results were also consistent with a higher risk of the vascular diseases myocardial infarction and angina in aromatase inhibitor users compared with tamoxifen users, but there was also a suggestion that this may be partly driven by a protective effect of tamoxifen on these outcomes. Data were limited, and evidence was generally inconsistent for all other cardiovascular disease outcomes.

**Conclusion:**

This review has collated substantial randomised controlled trial and observational evidence on the effect of endocrine therapies on several specific cardiovascular disease outcomes including venous thromboembolism and myocardial infarction, progressing knowledge. Although the choice of aromatase inhibitor or tamoxifen will primarily be based on the effectiveness against the recurrence of breast cancer, this review shows that the individual patient’s risk of venous or arterial vascular disease should be an important secondary consideration.

**Systematic review registration:**

Prospero CRD42017065944.

## Introduction

Endocrine therapies—namely, tamoxifen and aromatase inhibitors—reduce the risk of reoccurrence of breast cancer in patients diagnosed as having oestrogen receptor and/or progesterone receptor positive breast cancer following surgery (adjuvant treatment). The efficacy of tamoxifen, irrespective of menopausal status, has been confirmed in several randomised controlled trials,^1^ but UK guidelines were changed in 2006 to reflect the evidence that aromatase inhibitors are more efficacious in postmenopausal women.^2^ Concerns exist that endocrine therapies could increase the risk of cardiovascular disease—for example, through suppression of the cardiovascular protective effects of oestrogens.^3^ With improved survival after breast cancer, cardiovascular disease has become an increasingly important source of long term morbidity and mortality among breast cancer survivors.^4^ Understanding any associations between treatment of cancer and risk of cardiovascular disease is critical to inform prevention and management of adverse cardiovascular effects.

Several systematic reviews and meta-analyses of randomised controlled trials,^2^ ^5^ ^6^ ^7^ ^8^ ^9^ and some non-systematic reviews,^10^ ^11^ ^12^ ^13^ have compared cardiotoxicities of endocrine therapies in breast cancer survivors (systematic reviews summarised in appendix 1). Several reported a higher incidence of adverse cardiovascular disease outcomes in users of aromatase inhibitors compared with tamoxifen, but results were not universally in agreement. The most recent meta-analysis suggested a 19% higher risk of a composite of cardiovascular disease outcomes, excluding venous thromboembolism, in users of aromatase inhibitors compared with tamoxifen but hypothesised that this may reflect the cardioprotective effects of tamoxifen.^5^ Important limitations of the randomised controlled trial evidence included in these reviews may have contributed to the mixed picture, including high degrees of trial heterogeneity, limited power of individual trials, and inconsistent reporting of cardiovascular disease outcomes in trials focusing on anticancer effects. Previous reviews have also mainly reported results for composite cardiovascular disease outcomes rather than clinically specific cardiovascular diseases and omitted the growing body of evidence from observational studies on this topic, which often include large study populations in real world settings and longer follow-up.

The aims of this systematic review were to identify and summarise both randomised controlled trial and observational evidence on associations between endocrine therapies and a wide range of specific clinical cardiovascular disease outcomes in women with a history of early breast cancer, to describe the differences between findings from randomised controlled trials and real world observational studies, and to assess the quality and potential for bias in studies investigating this topic.

## Methods

### Inclusion criteria

We included randomised controlled trials and observational studies if they carried out at least one analysis assessing the risk of a specific cardiovascular disease outcome associated with tamoxifen, aromatase inhibitors, or a comparison of the two treatments after the diagnosis of non-metastatic breast cancer in women. The outcomes of interest were vascular disease—angina, myocardial infarction, revascularisation procedures, sudden cardiac arrest, stroke (haemorrhagic and ischaemic), and peripheral vascular disease; myocardial disease—cardiomyopathy, heart failure, and arrhythmia; venous thromboembolism; pericarditis; and valvular heart disease.

We excluded studies if only a composite cardiovascular disease outcome or mortality from cardiovascular disease was assessed, only women with metastatic breast cancer were included in the study population, or the study exclusively analysed temporal differences for the same treatment on the risk of cardiovascular disease. We also excluded previous systematic reviews and meta-analyses exploring the cardiotoxicities of systemic breast cancer therapies (specifically endocrine therapies), but we included relevant randomised controlled trials captured in these reviews that were not captured in the main search, along with any more recent or previously unidentified trials. We also manually searched all randomised controlled trials of endocrine therapy for breast cancer published since the most recent systematic review to ensure that more recent trial papers were not missed.

### Search strategy and data extraction

We used the health and medical literature databases Medline and Embase to search for relevant publications. The searches were performed in June 2018. Conference abstracts, grey literature, and unpublished studies were not included. To identify all relevant literature, the search strategy for each database included a comprehensive list of both index and free text terms for breast cancer, endocrine therapies, and cardiovascular disease. The full search terms used are outlined in appendix 2. We manually searched the reference lists of all studies identified in the search to further identify relevant studies that were originally missed.

We extracted relative risks, odds ratios, or hazard ratios if they were calculated in the paper. We calculated the relative risk and 95% confidence interval if effect estimates were not presented but data on the number of outcome events in follow-up allowed their calculation. We also collated information on the country in which the study was based, study type (randomised controlled trial or observational), data source (if an observational study), study design (if an observational study), age of included patients, inclusion criteria, exclusion criteria, intervention arm (and number of patients in the arm), reference arm (and number of patients in the arm), primary endpoint, cardiovascular disease outcome(s) assessed, mean/median follow-up time, statistical methods, and covariates adjusted for.

One researcher (AM) identified all relevant studies from the original literature search and extracted the results of these studies. The study identification process was repeated by a combination of two other authors (RF and HS). The extraction table was also piloted on two studies by two researchers (AM and RF) to check reproducibility of key information.

### Risk of bias assessment

We used the Cochrane Collaboration’s tool for assessing risk of bias to assess risk of bias in randomised controlled trials.^14^ We then adapted this tool to produce separate risk of bias assessments for cohort and case-control studies, with domains for each type of bias that could be encountered within both observational study designs (appendices 3 and 4).

### Statistical analysis

We organised seven possible comparisons between study arms/exposures during follow-up into three groups defined a priori to aid presentation. Group 1 included the direct comparison between aromatase inhibitor use and tamoxifen use during follow-up. Group 2 included three comparisons, all characterised as addition of tamoxifen in the intervention arm during follow-up (tamoxifen versus placebo, tamoxifen versus no tamoxifen, sequenced therapy (tamoxifen followed by aromatase inhibitor or vice versa) versus aromatase inhibitor). Group 3 also included three comparisons, with addition of aromatase inhibitor in the intervention arm during follow-up (aromatase inhibitor versus placebo, aromatase inhibitor versus no aromatase inhibitor, sequenced therapy versus tamoxifen).

To investigate differences in study findings for the same cardiovascular disease outcome, study type (randomised controlled trial or observational), and comparison (for the seven possible comparisons outlined above), we used I^2^ tests and P values for Cochrane Q tests to assess heterogeneity.^15^ We considered an I^2^ value of above 25% to be evidence of between study heterogeneity.^16^ We used a fixed effect meta-analysis to combine individual study effects estimates if there was more than one study and no evidence of heterogeneity within cardiovascular disease outcome, study type, and comparison strata. If we found evidence of between study heterogeneity, we assessed studies within the same strata for differences in study population, statistical analysis methods, and covariate adjustments, but they were not meta-analysed. For the purposes of exploring heterogeneity and meta-analysing results, we considered randomised controlled trials and observational studies separately. We used a test for funnel plot asymmetry to examine publication bias if there were more than 10 studies within the same cardiovascular disease outcome, study type, and comparison strata.^17^

### Patient involvement

No patients were involved in setting the research question or the outcome choices, nor were they involved in developing plans for design or implementation of the study. No patients were asked to advise on interpretation or writing up of results.

## Results

**Fig 1:**
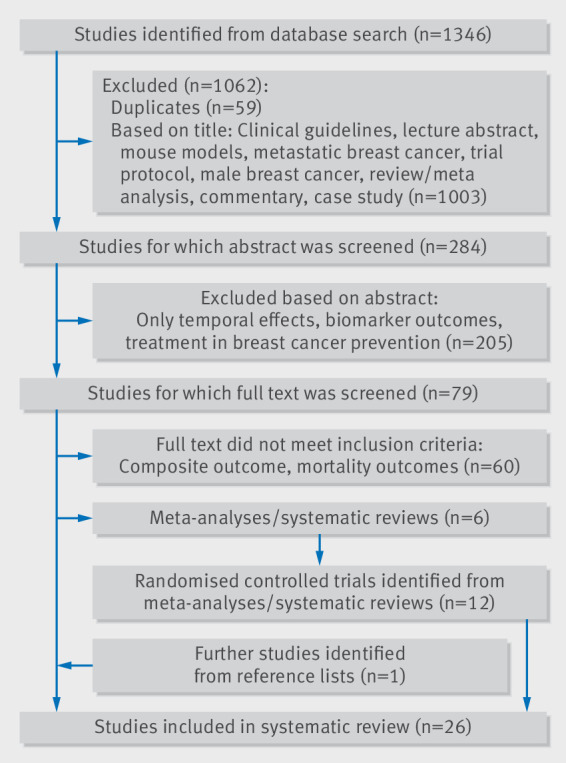
Flow diagram of screening process of studies included in systematic review. RCT=randomised controlled trial

Figure 1 outlines the screening process. We included 26 studies after applying the inclusion and exclusion criteria.^18^ ^19^ ^20^ ^21^ ^22^ ^23^ ^24^ ^25^ ^26^ ^27^ ^28^ ^29^ ^30^ ^31^ ^32^ ^33^ ^34^ ^35^ ^36^ ^37^ ^38^ ^39^ ^40^ ^41^ ^42^ ^43^ ^44^ Six previous meta-analyses or systematic reviews of randomised trials were also identified.^2^ ^5^ ^6^ ^7^ ^8^ ^9^ We identified 12 individual randomised controlled trials that met the inclusion criteria from within these meta-analyses and included them in our review. One further study was identified from scanning the reference lists of the other papers. The final 26 included studies consisted of 15 randomised controlled trials and 11 observational studies. Table 1 summarises the included studies, with a more detailed breakdown in appendix 5. There were minimal discrepancies between authors in the duplication of the search strategy.

**Table 1:** Overview of characteristics of studies included in systematic review. Values are numbers (percentages)

	Value (n=26)
**Study type**	
Randomised controlled trial	15 (58)
Observational	11 (42)
Case-control	4 (15)
Cohort	7 (27)
**Country/region**	
North America	8 (31)
Canada	2 (8)
USA	5 (19)
USA and Canada	1 (4)
Europe	11 (42)
Denmark	2 (8)
Germany	1 (4)
Italy	1 (4)
Scotland	1 (4)
Sweden	1 (4)
UK	3 (12)
Europe-wide	2 (8)
Rest of world	3 (12)
Taiwan	2 (8)
Egypt	1 (3)
International	4 (15)
**Study population**	
<80 years old	1 (4)
<70 years old	1 (4)
35-70 years old	1 (4)
45-69 years old	2 (8)
All women	7 (27)
Postmenopausal	13 (50)
Premenopausal	1 (4)
**Year of study**	
Before 2000	4 (15)
2000-10	13 (50)
After 2010	9 (35)
**Outcomes** *	
Vascular disease	
Myocardial infarction	14 (54)
Stroke	12 (46)
Angina	4 (15)
Peripheral vascular disease	1 (4)
Myocardial disease	
Heart failure	4 (15)
Arrhythmia	1 (4)
Thromboembolic events	15 (58)

* Individual studies often included more than one outcome.

The most commonly investigated outcomes were venous thromboembolism (n=15), myocardial infarction (n=14), and stroke (n=12). Arrhythmia and peripheral vascular disease were each investigated in a single study. Studied outcomes did not include revascularisation, sudden cardiac arrest, cardiomyopathy, pericarditis, or valvular heart disease.

### Bias assessment

Table 2 and table 3 show an overview of the risk of bias assessment of all randomised controlled trials and observational studies, with more detailed information in appendices 6 and 7. The main problem when assessing bias in randomised controlled trials was the incomplete reporting of methods, which in many cases made fully judging whether studies were prone to certain biases impossible. Three of the 15 randomised controlled trials were open label trials, and so were at higher risk of performance bias. Only one randomised controlled trial reported sufficient information to assess potential selective reporting of cardiovascular disease outcomes.

**Table 2:** Risk of bias assessment overview: observational studies

Paper	Study design	Exposure definition	Outcome/case definition	Control selection	Confounding	Missing data	Censoring
Abdel-Qadir 2016	Cohort	High	High	NA	Low	Unknown	Low
Chen 2014	Cohort	High	Low	NA	High	Unknown	Low
Haque 2016	Cohort	High	Low	NA	Low	Low	Low
Hernandez 2008	Cohort	Unknown	Low	NA	Low	Unknown	Low
Hernandez 2009	Cohort	Unknown	Low	NA	Low	High	Low
Ligibel 2012	Cohort	High	Low	NA	High	Unknown	Low
Yang 2014	Cohort	High	Low	NA	High	Unknown	Unknown
Bradbury 2005	Case-control	High	High	Low	High	Low	NA
Geiger 2004	Case-control	Low	Low	Low	High	High	NA
Geiger 2005	Case-control	Low	Low	Low	High	High	NA
Meier 1998	Case-control	Low	Low	Low	High	High	NA

NA=not applicable

**Table 3:** Risk of bias assessment overview: randomised controlled trials

Paper	Random sequence generation	Allocation concealment	Blinding	Incomplete outcome data	Selective reporting	Other sources of bias
Bliss 2012	Low	Unknown	Low	Low	Low	Low
Boccardo 2006	Unknown	Unknown	Unknown	Low	Unknown	Low
Coombes 2007	Low	Unknown	Low	Low	Unknown	Low
Fisher 1999	Unknown	Unknown	Unknown	Low	Unknown	Low
Fisher 2001	Low	Unknown	Unknown	Low	Unknown	Low
Forbes 2008	Low	Low	Unknown	Unknown	Unknown	Low
Jakesz 2005	Low	Low	High	Unknown	Unknown	Low
Kaufmann 2007	Low	Low	Unknown	Low	Unknown	Low
McDonald 1995	Unknown	Unknown	Unknown	Unknown	Unknown	Low
Colleoni 2011	Low	Unknown	Low	Low	Unknown	Low
Rutqvist 1993	Unknown	Unknown	Unknown	Low	Unknown	Low
van de Velde 2001	Low	Low	High	Unknown	Unknown	Low
Abo-Touk 2010	Low	Unknown	Unknown	Low	Unknown	Low
Goss 2005	Low	Unknown	Low	Low	Unknown	Low
Pagani 2014	Low	Unknown	High	Low	Unknown	Low

All observational studies had at least one domain categorised as being at high risk of bias. Six out of the 11 studies had a high risk of bias for the methods used to define exposure, which was mostly owing to not requiring women to have a minimum exposure period or several prescriptions before being categorised as exposed, raising the possibility of exposure misclassification. Risk of bias due to residual confounding was also present across both cohort and case-control observational studies. Seven studies adjusted only for basic risk factors and did not consider cardiovascular disease related treatment, cancer severity at diagnosis, or other cancer treatments such as chemotherapy or biological therapy.

We did not assess publication bias because no cardiovascular disease outcome, study type, or comparison strata included more than 10 studies.

### Vascular disease

**Fig 2:**
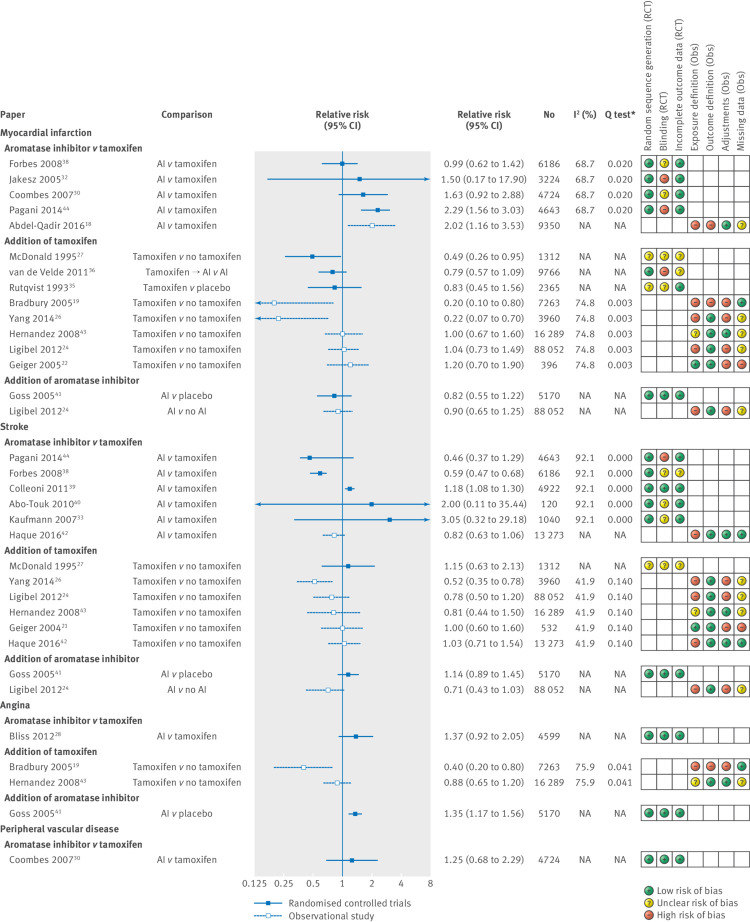
Estimated relative risk (95% CI) for studies examining use of endocrine therapy and risk of specific vascular diseases, with corresponding I^2^ tests, Q tests, and assessment of bias according to prespecified criteria. *P value. AI=aromatase inhibitor; NA=not applicable

Figure 2 shows relative risks and 95% confidence intervals for all vascular disease outcomes. Three of the four randomised controlled trials and one observational study that directly compared aromatase inhibitors with tamoxifen showed increased risks of myocardial infarction in the aromatase inhibitor group, with relative risks ranging from 1.50 to 2.29.^18^ ^30^ ^32^ ^38^ ^44^ However, the effect was statistically significant only in the observational study and one randomised controlled trial. Most (five out of eight) of the studies that explored the addition of tamoxifen observed a lower risk of myocardial infarction in the tamoxifen group,^19^ ^22^ ^24^ ^26^ ^27^ ^35^ ^36^ ^43^ including one trial and two observational studies that found a significantly protective relative risk. Although 12 studies explored the risk of stroke in users of endocrine therapy,^21^ ^24^ ^26^ ^27^ ^33^ ^38^ ^39^ ^40^ ^41^ ^42^ ^43^ ^44^ the picture was much more mixed and included estimates in both directions. Three of the five observational studies that compared tamoxifen use with non-tamoxifen use suggested a decreased risk of stroke in tamoxifen users.^21^ ^24^ ^26^ ^43^ Furthermore, the results for angina were consistent with patterns seen for myocardial infarction, but only four studies explored this outcome.^19^ ^28^ ^41^ ^42^ ^43^ However, one randomised controlled trial reported an increased risk of angina in aromatase inhibitor users compared with placebo users (relative risk 1.35, 95% confidence interval 1.17 to 1.56). Finally, only one inconclusive randomised controlled trial explored the risk of peripheral vascular disease in aromatase inhibitor users compared with tamoxifen users.^30^

### Myocardial disease

**Fig 3:**
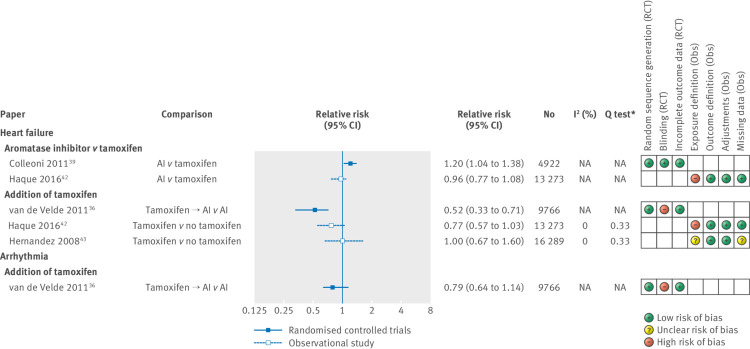
Estimated relative risk (95% CI) for studies examining use of endocrine therapy and risk of specific myocardial diseases, with corresponding I^2^ tests, Q tests and assessment of bias according to prespecified criteria

Figure 3 shows relative risks and 95% confidence intervals for all myocardial disease outcomes. One randomised controlled trial suggested an increased risk of heart failure in aromatase inhibitor users compared with tamoxifen users (aromatase inhibitor versus tamoxifen: relative risk 1.20, 1.04 to 1.38),^39^ but this was not replicated in an observational cohort study.^42^ A fixed effects meta-analysis based on two observational studies pointed towards a decreased risk of heart failure in tamoxifen users compared with non-users, albeit with a wide confidence interval (relative risk 0.84, 0.65 to 1.07; I^2^=0; Cochrane T test P=0.33; appendix 8),^42^ ^43^ which was replicated in a randomised controlled trial.^36^ One inconclusive study explored the risk of arrhythmia in tamoxifen users compared with non-users.^36^

### Venous thromboembolism

**Fig 4:**
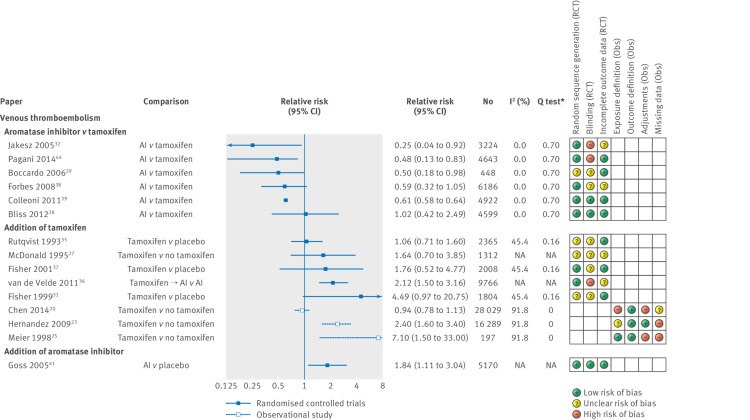
Estimated relative risk (95% CI) for studies examining use of endocrine therapy and risk of venous thromboembolism, with corresponding I^2^ tests, Q tests, and assessment of bias according to prespecified criteria

Figure 4 shows relative risks and 95% confidence intervals for all venous thromboembolism outcomes. Five out of six randomised controlled trials directly comparing the risk of venous thromboembolism in aromatase inhibitor users versus tamoxifen users estimated large protective relative risks ranging from 0.25 to 0.61,^28^ ^29^ ^32^ ^38^ ^39^ ^44^ with one further randomised controlled trial finding no association (I^2^=0%, Cochrane Q test P=0.70, fig 4). A fixed effects meta-analysis (appendix 9) suggested a decreased risk of thromboembolic events in aromatase inhibitor users compared with tamoxifen users (relative risk 0.61, 0.58 to 0.63). Five randomised controlled trials in which the key difference between treatment arms was use of tamoxifen reported an increased risk of thromboembolic events in the tamoxifen arm, with relative risks ranging from 1.06 to 4.49.^27^ ^31^ ^35^ ^36^ ^37^ The three randomised controlled trials comparing tamoxifen with placebo reported an increased risk of venous thromboembolism in tamoxifen users, but with 95% confidence intervals that crossed the null association (I^2^=45%; Cochrane Q test P=0.16).^31^ ^35^ ^37^ A further three observational studies compared the risk of thromboembolic events in tamoxifen users and non-tamoxifen users (I^2^=92%, Cochrane Q test P=0.00).^20^ ^23^ ^25^ Two found large increased risks in tamoxifen users (relative risks 2.40, 1.60 to 3.40, and 7.10, 1.50 to 33.00^23^ ^25^), and one reported no difference in risk of thromboembolic events (0.94, 0.78 to 1.13^20^), although this study had a high risk of bias owing to how exposure was defined. One randomised controlled trial reported an increased risk of thromboembolic events in aromatase inhibitor users compared with those given a placebo (relative risk 1.84, 1.11 to 3.04).^41^

## Discussion

Among 26 studies providing data on seven specific cardiovascular disease outcomes, we found consistent evidence of an increased risk of venous thromboembolism in tamoxifen users compared with non-users in both randomised controlled trials and observational studies, with a correspondingly decreased risk of venous thromboembolism when aromatase inhibitor users were compared directly with tamoxifen users. However, the direct effect of aromatase inhibitors on venous thromboembolism was less clear, as only a single randomised controlled trial compared aromatase inhibitor with placebo, finding an increased risk in aromatase inhibitor users. The evidence on the effects of endocrine therapies on vascular disease risks was mixed: most studies were consistent with a higher risk of myocardial infarction and angina in aromatase inhibitor users compared with tamoxifen users, and there was a suggestion that this may be partly driven by a protective effect of tamoxifen on these outcomes; inconsistent results were found for the associations with stroke. Of the few studies assessing other outcomes, data were limited and very mixed patterns were observed, making drawing conclusions difficult.

### Quality and limitations of evidence

Thirteen of the 15 randomised controlled trials identified disease-free survival as the primary outcome of the study, whereas all observational studies identified either one or several specific cardiovascular disease events as their primary outcome. Women with previous cardiovascular disease were therefore excluded from many observational studies but not from randomised controlled trials. Overall, women included in the randomised controlled trial populations were therefore likely to be at a higher absolute risk of cardiovascular disease during follow-up, which would be problematic only if the prevalence of cardiovascular disease at baseline was different between the treatment arms. In theory, randomisation should result in an equal prevalence of cardiovascular disease at baseline between arms. However, many included studies did not report information on randomisation and concealment of allocation, meaning that selection bias in relation to prevalent cardiovascular disease at the point of randomisation cannot be disregarded. Randomised controlled trials also did not report data on cardiovascular disease risk at baseline, but participants in trials are likely to be healthier than the general population and thus may have had less previous cardiovascular disease. As people with previous cardiovascular disease are likely to be underrepresented in randomised controlled trials, and are excluded from observational studies, the evidence on the association between endocrine therapies and risk of cardiovascular disease in this population remains limited. Furthermore, as the randomised controlled trials were mainly designed to assess disease-free survival, they were not always adequately powered to detect relative differences in the risk of clinical cardiovascular disease outcomes between treatment arms.

Definitions of cardiovascular disease outcomes were generally poorly recorded in the included studies. Between study variation could therefore exist in the measurement or coding of cardiovascular disease outcomes. Most oncology trials use the CTCAE criteria for adverse events, which have definitions that do not align with definitions in cardiology guidelines, although even the latter have variability. Furthermore, as observational studies rely on definitions of outcomes suggested by researchers and clinicians, differences in coding of outcomes could be a further source of heterogeneity in the observational studies. Without access to the outcome definitions and code lists used in these studies, fully understanding the extent to which the differences are problematic is challenging.

### Explanation of key findings

A biological rationale exists for the use of aromatase inhibitors increasing the risk of cardiovascular disease outcomes, as they reduce oestrogen concentrations and therefore the oestrogen-mediated protective effects on cardiovascular disease, such as regulation of serum lipid metabolism, increasing vasodilation, and inhibition of the development of atherosclerosis.^45^ Aromatase inhibitors could also increase the risk of hyperlipidaemia.^36^ Evidence from randomised controlled trials suggests that tamoxifen has cardioprotective effects by decreasing lipid concentrations.^46^ ^47^ This systematic review postulates that some evidence exists for aromatase inhibitor users having an increased risk of myocardial infarction, relative to women treated with tamoxifen. However, whether this is driven by a decreased risk of myocardial infarction in tamoxifen users or an increased risk in aromatase inhibitor users is unclear, as results on the individual effects of tamoxifen and aromatase inhibitor are inconclusive.

We found evidence of heterogeneity between all four strata of observational studies exploring the same exposure and outcome (risk of myocardial infarction, stroke, angina, and venous thromboembolism with the addition of tamoxifen), which was potentially driven by the differences in study populations, statistical techniques used, and covariates adjusted for (appendix 5). For example, one cohort and one case-control study reported an increased risk of venous thromboembolism in tamoxifen users compared with non-tamoxifen users in European study populations (Denmark and the UK, respectively), whereas a study in Taiwan reported no evidence of a difference in risk. More broadly, as this systematic review attempted to cover a wide range of clinical cardiovascular disease outcomes, some included observational studies focused on one cardiovascular disease outcome, whereas others covered a broad range of cardiovascular disease outcomes. Different statistical techniques and covariate adjustments were therefore needed. The effect of this heterogeneity between studies was witnessed in the varying relative risks reported within these strata of observational studies, which could be either a product of genuine discrepancies in risks between contrasting populations or the effect of residual confounding and different statistical techniques.

### Comparison with other studies

The addition of observational studies in this review allowed comparison of results between real world populations and randomised controlled trials that generally use homogeneous study populations. Overall, we mostly found agreement in the direction of effect between randomised controlled trials and observational studies, but several observational studies reported more extreme effect estimates in comparison with randomised controlled trials (the risk of myocardial infarction with the addition of tamoxifen and the risk of venous thromboembolism with the addition of tamoxifen). However, these observational studies all had a high risk of bias in at least two assessment of bias categories. Including observational studies also enabled further evidence to be gathered where little or no evidence from randomised controlled trials existed. For example, we identified six observational analyses of vascular endpoints finding good or strong evidence for a higher risk for aromatase inhibitor compared with tamoxifen or a lower risk for tamoxifen compared with no tamoxifen. Most randomised controlled trial analyses were underpowered to detect differences in vascular endpoints, with only three finding similar clear evidence despite several others being suggestive of associations in the same direction.

We grouped comparisons on the basis of the drug women were given at the beginning of follow-up. For example, in several randomised controlled trials, women were given two to three years of tamoxifen before being randomised to either aromatase inhibitor or the continuation of tamoxifen for a further two to three years, with follow-up beginning at the point of randomisation. We classed these as a direct comparison of aromatase inhibitor versus tamoxifen, whereas previous reviews classed these comparisons as sequenced therapy versus tamoxifen alone. As all women had had the same treatment regimen before randomisation, classing these as aromatase inhibitor versus tamoxifen was a reasonable comparison to make.

The most recent meta-analysis by Khosrow concluded that randomised controlled trials directly comparing aromatase inhibitors with tamoxifen suggest that aromatase inhibitors are associated with an increased risk of cardiovascular events, but the cardioprotective effects of tamoxifen may account for this increased risk.^5^ However, Khosrow et al used composite cardiovascular disease endpoints (excluding venous thromboembolism), which are defined slightly differently within each trial. We stratified cardiovascular disease events into more specific outcomes and found a similar pattern for several vascular cardiovascular disease outcomes. The results for other cardiovascular disease outcomes including heart failure suggest a similar trend, but few studies have specifically explored these outcomes, so definite conclusions are unattainable. Like our study, that of Khosrow et al was inconclusive about the effects of endocrine therapy on cerebrovascular events. Another recent review by Rydén reported, with a high quality of evidence, that the risk of venous thromboembolism was higher in tamoxifen users than aromatase inhibitor users in randomised controlled trials.^2^ Our study agrees with these results but also shows that this may be accounted for by the increased risk in tamoxifen users.

### Strengths and limitations of this review

This study focused on individual clinical cardiovascular disease outcomes, excluding studies that reported composite outcomes. Understanding the effect of endocrine therapies on cardiovascular disease as a whole has several advantages, such as the potential to change the modifiable risk factors weight, smoking, statin use, and alcohol intake, which are present across all clinical cardiovascular diseases. However, understanding the effect of endocrine therapies on more specific clinical cardiovascular disease outcomes has the potential to enable clinicians to be targeted in their approach to preventing these outcomes in breast cancer survivors. The only composite outcome that we explored was venous thromboembolism, as some studies in this group included only deep vein thrombosis outcomes whereas others also included pulmonary embolism within a venous thromboembolism outcome. However, this grouping is relevant owing to the clinical similarities of deep vein thrombosis and pulmonary embolism.

Some relevant studies may have been missed, as searches of literature database take into account only indexed key terms or words used in the title and abstract. Studies in which the secondary outcome was a cardiovascular disease would therefore not have been identified in the original search. For example, several randomised controlled trials that focused on anticancer efficacy do not mention cardiovascular diseases in the title, abstract, or indexed keywords. However, we searched multiple large databases, manually searched the included studies’ reference lists and relevant meta-analyses, and searched all endocrine therapy trials since the most recent meta-analysis, which was an indirect way of identifying the aforementioned randomised controlled trials and made this review as comprehensive as possible within the restricted framework imposed by the literature databases.

### Implications of findings

This review establishes the need for clinical vigilance and possible preventive measures when prescribing endocrine therapies to women at risk of venous thromboembolism. Knowledge has also been progressed on the effects of endocrine therapies on the risk of vascular cardiovascular diseases, for which little evidence previously existed. However, we also showed that little or no evidence is available on the effect of endocrine therapies on several other specific cardiovascular disease outcomes, although substantial trial evidence outlines the effect on cardiovascular diseases generally. This is unlikely to be studied in future randomised controlled trials, so it is vital that large observational studies are carried out with details of baseline cardiovascular disease risk and drug treatment and clear definitions of cardiovascular disease events to fully understand the effects that endocrine therapies have on potentially fatal cardiovascular disease outcomes such as myocardial infarction, stroke, and heart failure.

### Conclusions

Overall, the totality of the randomised controlled trial and observational evidence suggests a decreased risk of venous thromboembolism in aromatase inhibitor users compared with tamoxifen users, which is probably accounted for by an increased risk in tamoxifen users. The evidence also suggests that tamoxifen may have a protective association with vascular cardiovascular diseases, which may drive the higher risk of these outcomes in aromatase inhibitor users when directly compared with tamoxifen users. The results for some cardiovascular disease outcomes is still a mixed picture, many of the existing studies are susceptible to various sources of bias, and cardiovascular disease outcomes collected in oncology trials are generally limited. Nevertheless, the addition of observational studies alongside randomised controlled trials has substantially increased the amount of evidence supporting these conclusions. However, further high quality evidence is still needed for several cardiovascular disease outcomes. Although choice of aromatase inhibitor or tamoxifen will primarily be based on the effectiveness against recurrence of breast cancer, the individual patient’s risk of venous or arterial vascular disease is an important secondary consideration, and the totality of evidence we present will thus help to inform prescribing.

**What is already known on this topic**

Several meta-analyses of randomised controlled trials have reported the effect of endocrine therapies used in adjuvant treatment of breast cancer on the risk of composite cardiovascular disease outcomes
However, these reviews have not reported the effect of endocrine therapies on a range of clinically specific cardiovascular diseases
They have also omitted the growing body of evidence from observational studies, which often include large study populations in real world settings, as well as longer follow-up

**What this study adds**

Observational evidence is generally consistent with trial evidence reporting an increased risk of venous thromboembolism in tamoxifen users compared with both non-users and aromatase inhibitor users
Evidence also exists of a higher risk of vascular disease in aromatase inhibitor users compared with tamoxifen users, which may be driven by a protective effect of tamoxifen

## Web Extra

Extra material supplied by the author

Supplementary appendices

## Notes

Ethical approval: Not needed.

Data sharing: All data are freely available within the appendices. No additional data available.

Transparency: The lead author affirms that the manuscript is an honest, accurate, and transparent account of the study being reported; that no important aspects of the study have been omitted; and that any discrepancies from the study as planned (and, if relevant, registered) have been explained.
