# Supplementary material for: Long term adjuvant endocrine therapy and risk of cardiovascular disease in female breast cancer survivors: systematic review
Source: BMJ. 2018 Oct 8;363:k3845. doi: 10.1136/bmj.k3845 (PMC6174332; doi:10.1136/bmj.k3845)

APPENDICES

Appendix 1 – Overview of previous meta-analyses

Author	Year	Inclusion criteria	Main comparison	Main Cardiovascular Outcomes	Meta-analysis result RR (95% CI)	Notes
Khosrow-Khavar	2016	Phase III RCTs examining third generation AIs and tamoxifen among post-menopausal women with a diagnosis of breast cancer, with CVD or cerebrovascular adverse events reported.	AI vs Tam	CVD events	1.19 (1.07-1.34)	Result from AI vs Tam monotherapy. Concluded that the cardio-protective effects of tamoxifen accounted for the increase in CVD risk. Also explored sequenced therapy.
				Cerebrovascular events	0.96 (0.61-1.51)	
Ryden	2016	RCTs with long-term (at least 5 years) follow-up data of AI compared to tamoxifen or placebo with either efficacy (DFS and OS) or side effect outcomes	AI vs Tam	CVD events	1.13 (0.96-1.33)	Result from AI vs Tam monotherapy. Only one study in AI vs Tam analysis. Also explored sequenced therapy, and looked at time on and off treatment
Aydiner	2013	RCTs that included postmenopausal women that had undergone surgery for estrogen-sensitive early breast cancer, and examined the comparative effects of AIs and tamoxifen (either as monotherapy, sequenced therapy, or extended therapy) in relation to efficacy outcomes	AI vs Tam	CVD events	1.23 (0.95-1.60)	Result from AI vs Tam monotherapy. Also explored sequenced therapy.
				Thromboembolic events	0.61 (0.47-0.80)	
Amir	2011	Phase III RCTs that compared AIs with tamoxifen as initial adjuvant therapy in postmenopausal women with early stage breast cancer. Only trials that had treatment durations longer than 5 years were included.	AI vs Tam	CVD events (including MI, angina, and cardiac failure)	1.26 (1.10-1.43)	Result includes direct AI vs Tam, Tam to AI vs AI alone, and tam to AI vs AI alone
				Cerebrovascular events (including cerebrovascular accident and transient ischemic attack)	1.01 (0.81-1.26)	
				Venous Thrombosis (any venous thromboembolic episode)	0.55 (0.46-0.64)	
Cuppone	2007	Phase III RCTs that explored the cardiovascular risk of adjuvant AI compared to tamoxifen as an early switch strategy (after 2-3 years tamoxifen) or as an upfront strategy (starting at the time of surgery and planned for 3 years. All trails must have included women who were previously untreated and had undergone surgical resection for early breast cancer.	AI vs Tam	CVD events	1.30 (1.07-1.60)	Result Includes both upfront and early switch comparisons of AI and tamoxifen
				Thromboembolic events	0.53 (0.42-0.65)	
				Cerebrovascular events	0.84 (0.68-1.05)	
Braithwaite	2003	Breast cancer treatment RCTs that explored the effect of tamoxifen on vascular outcomes	Tam vs No tam/ placebo	MI	0.74 (0.47-1.16)	Also explored some outcomes in trials of post-menopausal women, breast cancer reduction trials, and trials with tamoxifen as only treatment
				Stroke	1.48 (1.07-2.04)	

Appendix 2 - Systematic review search strategy

	**Medline**
Breast Cancer	
MeSH terms	breast neoplasms or carcinoma, ductal, breast or carcinoma, lobular or inflammatory breast neoplasms or unilateral breast neoplasms or triple negative breast neoplasms
Keywords	breast cancer or breast neoplasm* or breast tumour or breast adenocarcinoma or breast carcinogenesis or breast carcinoma or breast sarcoma
Endocrine Therapy	
MeSH terms	tamoxifen or aromatase inhibitors
Keywords	tamoxifen or aromatase inhibitor* or anastrazole or exemestane or letrozole or endocrine therapy
Cardiovascular Disease	
MeSH terms	cardiovascular diseases or heart diseases or cardiotoxicity or coronary artery disease or cardiomyopathies or heart arrest or heart failure or heart failure, diastolic or heart failure, systolic or heart valve diseases or aortic valve insufficiency or aortic valve stenosis or mitral valve insufficiency or mitral valve stenosis or pulmonary valve insufficiency or pulmonary valve stenosis or tricuspid valve insufficiency or tricuspid valve stenosis or angina pectoris or angina, unstable or angina, stable or myocardial infarction or stroke or venous thromboembolism or pulmonary embolism or pericarditis or peripheral vascular disease or arrhythmias, cardiac
Keywords	cardiovascular* or CVD or cardiac or cardiotoxi* or heart disease* or coronary artery dis* or revascular* or coronary bypass or artery bypass or aorta bypass or cardiomyopathy* or cardiopulmonary arrest* or cardiac arrest* or heart arrest* or heart failure or valvular*disease or valve disease or valve stenosis or valve insufficiency or angina* or heart infarc* or myocardial infarc* or heart attack or coronary infarc* or stroke or tia or transient ischemic attack or cerebrovascular accident or venous thromboembolism or deep*thrombo* or thromboem* or pulmonary embolism or pericarditis or peripheral vascular or peripheral art* or arrhythmia* or fibrillation or heart*flutter
Limits	
	• English language<br>• Humans<br>• 1960 –Current year
	**Embase**
Breast Cancer	
Indexed terms	breast cancer or breast tumour or basal like breast cancer or breast adenocarcinoma or breast carcinogenesis or breast carcinoma or breast sarcoma or estrogen receptor positive breast cancer or inflammatory breast cancer or triple negative breast cancer
Keywords	breast cancer or breast neoplasm* or breast neoplasm or breast tumour or breast adenocarcinoma or breast carcinogenesis or breast carcinoma or breast sarcoma
Endocrine Therapy	
Indexed terms	aromatase inhibitor or anastrozole or exemestane or letrozole or tamoxifen
Keywords	chemotherapy or anthracycline or daunorubicin or doxorubicin or epirubicin or cyclophosphamide or fluorouracil or methotrexate or taxoid* or taxane* or paclitaxel or docetaxel or tamoxifen or aromatase inhibitor* or anastrazole or exemestane or letrozole or endocrine therapy or trastuzumab or Herceptin or breast cancer treatment
Cardiovascular Disease	

Indexed terms	heart disease/ or cardiovascular disease/ or cardiotoxicity/ or heart arrhythmia/ or heart atrium arrhythmia/ or heart ventricle arrhythmia/ or atrial fibrillation/ or heart atrium flutter/ or heart ventricle arrhythmia/ or heart ventricle flutter/ or heart ventricle fibrillation/ or heart fibrillation/ or heart failure/ or acute heart failure/ or congestive heart failure/ or diastolic heart failure/ or systolic heart failure/ or heart ventricle failure/ or heart left ventricle failure/ or heart right ventricle failure/ or Ischemic cardiomyopathy/ or cardiomyopathy/ or congestive cardiomyopathy/ angina pectoris/ or stable angina pectoris/ or unstable angina pectoris/ or heart infarction/ or acute heart infarction/ or heart atrium infarction/ or pericarditis/ or valvular heart disease/ or aorta valve disease/ or mitral valve disease/ or pulmonary valve disease/ or tricuspid valve disease/ or aorta valve stenosis/ or mitral valve stenosis/ or heart valve stenosis/ or pulmonary valve stenosis/ or tricuspid valve stenosis/ or revascularization/ or heart arrest/ or cardiopulmonary arrest/ or cerebrovascular accident/ or venous thromboembolism/ or deep vein thrombosis/ or thromboembolism/ or embolism/ or vein thrombosis/ or peripheral vascular disease/
Keywords	cardiovascular* or CVD or cardiac or cardiotoxi* or heart disease* or coronary artery dis* or arrhythmia* or fibrillation or heart*flutter or heart failure or cardiomyopathy or angina or heart*infarc* or myocardial infarc* or heart attack or coronary infarc* or pericarditis or valvular*disease or valve disease or valve stenosis or valve insufficiency or revascular* or coronary bypass or artery bypass or aorta bypass or cardiopulmonary arrest* or cardiac arrest* or heart arrest* or cerebrovascular accident or stroke or tia or transient ischaemic attack or venous thromboembolism or deep*thrombo* or thromboem* or pulmonary embolism or peripheral vascular or peripheral art*
Limits	
	• English language<br>• Human<br>• Embase<br>• 1960 –Current year<br>• Article or review

Appendix 3 - Bias assessment criteria – cohort studies

Exposure	**Low**<br>• Minimum exposure period or need for several prescriptions before classified<br>• Exposure ascertained through prescription or pharmacy records<br>**High**<br>• Exposure ascertainment not clearly defined, or defined by patient or physician recall<br>• Future information used to inform exposure status at baseline<br>• Potential for exposure misclassification due to no information of exposure prior to index<br>• No minimum exposure period or need for several prescriptions<br>• Non exposed or referent group from a different population to exposed
Outcome Assessment	**Low**<br>• Well defined diagnosis using hospital records, GP diagnosis, or similar methods<br>• Method of outcome ascertainment has been clearly validated<br>**High**<br>• Unclear method of diagnosis, or diagnosis defined by patient or physician recall<br>• Potential for differential misclassification due to different methods of outcome ascertainment being used for different exposure groups
Adjustments	**Low**<br>• IPTW adjustment for CVD risk factors, CVD related treatment, cancer severity, major non-CVD comorbidities, other cancer treatments<br>• Adjustment for most or all of the risk factors outlined above at baseline<br>**High**<br>• Minimal adjustment for one or two of the risk factors outlined above at baseline<br>• No adjustment
Missing data	**Low**<br>• None or low percentage of missing data, or appropriate missing data technique used such as multiple imputation<br>**High**<br>• Substantial amount of missing data (>20%) with no methods applied to deal with missingness<br>• A missing category fitted to deal with missing data
Censoring	**Low**<br>• No censoring/loss to follow up<br>• Appropriate method of adjustment or sensitivity analysis if censoring or loss to follow up present<br>• Censoring unlikely to have impact on results<br>**High**<br>• No adjustment or additional analysis where censoring/loss to follow up may cause bias

Appendix 4 - Bias assessment criteria – case-control studies

Case Definition	**Low**<br>• Well defined diagnosis using hospital records, GP diagnosis, or similar methods<br>• Method of case definition ascertainment has been clearly validated<br>**High**<br>• Unclear method of diagnosis, or diagnosis defined by patient or physician recall<br>• Potential for differential misclassification due to different methods of case ascertainment between exposure groups<br>• Likely that outcome can occur at time that is not appropriate to risk period relative to exposure
Control Selection	**Low**<br>• Controls comparable to and chosen from the same population as cases<br>**High**<br>• Controls systematically different to cases due to being selected from a different population, or have very different characteristics that have not been adjusted for
Exposure Assessment	**Low**<br>• Exposure ascertained through prescription or pharmacy records<br>• Same method for exposure ascertainment used for cases and controls<br>**High**<br>• Exposure ascertainment not clearly defined, or defined by patient or physician recall<br>• Risk of misclassification due to incomplete records on past exposure<br>• Potential for misclassification of exposure based on outcome, or different methods used for exposure ascertainment between cases and controls
Adjustments	**Low**<br>• Detailed adjustment for CVD risk factors, CVD related treatment, cancer severity, major non-CVD comorbidities, other cancer treatments<br>• Adjustment for most or all of the risk factors outlined above, in less detail<br>**High**<br>• Minimal adjustment for one or two of the risk factors outlined above<br>• No adjustment<br>• Risk factors ascertained through recall by patient or physician
Missing data	**Low**<br>• None or low percentage of missing data, or appropriate missing data technique used<br>**High**<br>• Substantial amount of missing data (>20%) with no methods applied to deal with missingness<br>• A missing category fitted to deal with missing data

Appendix 5 – Overview of included studies

**Author**	Meier	Geiger	Bradbury
Year	1998	2004	2005
Title	Tamoxifen and risk of idiopathic venous thromboembolism	Stroke risk and tamoxifen therapy for breast cancer	Tamoxifen-treated breast carcinoma patients and the risk of acute myocardial infarction and newly-diagnosed angina
Country	UK	USA	UK
Study Type	Observational	Observational	Observational
Data source	GPRD	Kaiser Permanente Southern California	GPRD
Study Design	Case control	Case control	Nested case control
Age	<70 (at time of outcome)	All patients	35-80 years old
Inclusions	Women who had a computer-recorded diagnosis of breast cancer in or after 1980 and who were hospitalised for a first-time diagnosis of deep vein thrombosis or pulmonary embolism between January 1, 1991 and December 31, 1996. For each case, up to 10 control women with breast cancer were randomly selected, matched on age (within two years), duration of breast cancer (same year of breast cancer) and calendar year of VTE (same index date). Women were ineligible to be controls if they had—according to the computerized medical record—recurrent or metastatic breast cancer, died within 6 month after the index date, or underwent mastectomy, chemotherapy, radiotherapy, trauma, or major surgery within 6 months prior to the index date.	All women with a first invasive breast cancer diagnosed at KPSC between January 1, 1980, and July 1, 2000.	Women with a first-time diagnosis of breast carcinoma who were treated with tamoxifen or with bladder carcinoma, colorectal carcinoma, or non-melanoma skin cancer between January 1, 1991 and December 31, 1999. Women with other cancers (bladder, colorectal, and non-melanoma skin cancer), were selected to provide an unexposed population, because most women with breast carcinoma in the GPRD were treated with tamoxifen, and to increase the comparability of the exposure reference group to the tamoxifen-exposed group with respect to ongoing medical surveillance.
Exclusions	Any other malignancies besides breast cancer, a history of VTE or thrombophlebitis, stroke, angina pectoris, myocardial infarction, diabetes mellitus, chronic renal disease, hypertension, hyperlipidemia, intermittent claudication, systemic lupus erythematosus, epilepsy, connective tissue disorders or cystic fibrosis. Furthermore all potential cases were excluded if they underwent mastectomy, chemotherapy, radiotherapy, trauma (i.e. accident, bone fracture) or major surgery (i.e. abdominal surgery, hip replacement) within 6 months prior to the index date, who had recurrent or metastatic breast cancer, or who were in their terminal phase and died within 6 months after the index date (subjects who died from pulmonary embolism were included).	Patients with a subsequent primary cancer diagnosis (other than a second primary breast cancer, cervical cancer in situ, or basal or squamous cell skin cancer) before their stroke diagnoses were excluded from the study because the other cancer could alter their breast cancer treatment or their stroke risk. Patients with thromboembolic disease diagnoses other than stroke (i.e., myocardial infarction, venous thromboembolism, or pulmonary embolism) were excluded.	Women were excluded if they had a history of cancer, MI, angina pectoris, congestive heart failure, or HIV/acquired immunodeficiency syndrome before the study entry date. Women with known HIV infection were excluded because HIV infection may complicate cancer therapy, including adjuvant therapy. Women were required to have at least 1 year of recorded follow-up after their study entry date to assure adequate follow-up.
Intervention arm	Tamoxifen (any, currently exposed in VTE case-control analysis at index date) (n=133)	Any tamoxifen (in stroke case-control analysis) (n=286)	Current tamoxifen. Women who received 2 or more tamoxifen prescriptions within 1 year of their index date were considered current users (n=49)
Reference arm	Never or past tamoxifen (in VTE case-control analysis at index date) (n=64)	Unexposed to tamoxifen (in stroke case-control analysis). Unlikely to have been prescribed AIs due to study period being before approval of AIs (n=246)	Unexposed to tamoxifen. Unlikely to have been prescribed AIs due to study period being before approval of AI (n=158)

Primary end point	VTE	Stroke (hospitalisation)	Ischaemic heart disease diagnosis in primary care
Follow up time	Mean follow up 49.2 months (range 12-144)	Mean at-risk period 68.4 months (standard deviation 54 months)	N/A
Statistical methods (if available for CVD outcome)	A matched analysis was conducted by using conditional logistic regression models, and relative risk estimates (odds ratios) of developing VTE with regard to current and past use were obtained, using never users as reference group.	Case patients were compared with their individually matched control subjects using univariate and multivariable conditional logistic regression methods. Crude and adjusted odds ratios were estimated, and 95% confidence intervals were calculated. These analyses were limited to case patients who had their first stroke after their breast cancer diagnosis and their matched control subjects.	The risk of IHD was assessed for current tamoxifen users and according to the dose-response measures among all cases combined and among cases stratified by diagnosis of angina or MI. Odds ratios and 95% CIs were estimated using conditional logistic regression modeling
Adjustments	Cases and controls matched on age (within two years), duration of breast cancer (same year of breast cancer) and calendar year of VTE (same index date). Then analyses adjusted for BMI (< 30, 30+ kg m−2, unknown), smoking status (never, ex, current, unknown), and hysterectomy status (yes, no)	Menopausal status (pre- or perimenopausal, naturally postmenopausal, or menopausal because of surgery); history of hypertension (no, yes but not requiring medication, and yes requiring medication); history of diabetes (no, yes but not requiring medication, and yes requiring medication); chemotherapy (yes, no)	Cases and controls were matched on the date of the case's IHD diagnosis, age (1 year), and study entry date (6 months). Analyses were further adjusted for BMI (kg/m2), treated hypertension, use of hormone replacement therapy, and smoking status. Information concerning these risk factors was ascertained from the data base on or before the index date
Relative risk taken from paper, or calculated from raw numbers	Paper	Paper	Paper
CVDs outcome(s)	Thromboembolic events	Stroke	Angina, MI

**Author**	Geiger	Hernandez	Ligibel
Year	2005	2009	2012
Title	Myocardial infarction risk and tamoxifen therapy for breast cancer	Tamoxifen treatment and risk of deep venous thrombosis and pulmonary embolism: a Danish population-based cohort study	Risk of myocardial infarction, stroke, and fracture in a cohort of community-based breast cancer patients
Country	USA	Denmark	USA
Study Type	Observational	Observational	Observational
Data source	Kaiser Permanente Southern California	Danish Registries	HealthCore Integrated Research Database
Study Design	Case control	Cohort	Cohort
Age	All patients	45-69 years old	Post-menopausal
Inclusions	All women with a first invasive breast cancer diagnosed at KPSC between January 1, 1980, and July 1, 2000.	Women eligible for the study were diagnosed with International Union Against Cancer stage I or stage II estrogen receptor-positive breast cancer between 1990 and 2004 at ages 45 to 69 years, as reported to the Danish Breast Cancer Cooperative Group (DBCG) clinical database	Women who were enrolled a minimum of 6–12 months before the first of at least 2 diagnosis codes for breast cancer during 2001–2007 and women with no diagnosis codes for breast cancer who were used as controls
Exclusions	Patients with another cancer diagnosis (other than second primary breast cancer, cervical cancer in situ or basal or squamous cell skin cancer) or thromboembolic disease (stroke, venous thromboembolism or pulmonary embolism) occurring before their MI were excluded	Women with no existing cardiovascular disease (defined using ICD-8 and ICD-10 codes) as of the date of breast cancer surgery	Metastatic cancer
Intervention arm	Any tamoxifen (in MI case-control analysis) (n=216)	Any tamoxifen during follow up (n=8232)	Currently exposed to Tamoxifen (n=4710) or AI (n=9067). Patients who were simultaneously prescribed both drugs contributed analysis time to both the tamoxifen and AI group.
Reference arm	Unexposed to tamoxifen (in case-control analysis). Unlikely to have been prescribed Ais due to study period being before approval of AIs (n=165)	Unexposed to tamoxifen. Unlikely to have been prescribed AIs due to study period being before approval of AIs (n=8057)	Not currently exposed to either tamoxifen or AI therapy (n=29497)
Primary end point	Myocardial infarction (hospitalisation)	DVT/PE (ICD-8 and -10 codes 45,099; 45,100; DI260; DI269; DI269A; DI801; DI802; DI802B; DI803; and DI803E)	Myocardial infarction, ischemic stroke, and fractures
Follow up time	Mean at-risk period 64.4 months (standard deviation 58.8 months)	Median follow up 48 months (range 0-174)	Median follow up 30 months for breast cancer patients and 33.5 months for non-breast-cancer patients
Statistical methods (if applicable/available for CVD outcome)	Case patients were compared with their individually matched control subjects using univariate and multivariable conditional logistic regression methods. Crude and adjusted odds ratios were estimated, and 95% confidence intervals were calculated. These analyses were limited to case patients who had their first stroke after their breast cancer diagnosis and their matched control subjects.	Follow-up was initiated 3 months after the surgery date. Follow-up ended on December 31, 2005. Risks of events were analyzed individually by year for the first 5 years of follow-up, and then cumulatively for Years 1 to 5. RRs and 95% confidence intervals were calculated as estimates of the association between tamoxifen therapy and incident thromboembolic events. Cox proportional hazards models were used to estimate crude HRs and adjusted HRs controlling for confounding, for years 1 to 5 individually, and for Years 5 to 10 taken together. the proportional hazards assumption was	Propensity score matching was used. Cox proportional hazards models with time varying treatment variables were used to assess whether treatment with AIs or tamoxifen was associated with MI and stroke among women with breast cancer and to assess the association of breast cancer with the outcomes of interest. The time-varying treatment variables allowed women to contribute information to the treatment group when on treatment and to the control group when not on treatment; women who received both AIs and tamoxifen contributed to both groups. For each outcome event, women were followed from the time of their first diagnosis code only

		tested by adding a covariate to the model to represent the interaction between exposure and the log of survival time	until the occurrence of the event or the censoring of their observation.
Adjustments	Menopausal status (pre- or perimenopausal, naturally postmenopausal, or menopausal because of surgery); history of hypertension (no, yes but not requiring medication, and yes requiring medication); history of diabetes (no, yes but not requiring medication, and yes requiring medication); chemotherapy (yes, no)	Age, surgical procedures (other than breast cancer surgery), metastatic tumors other than breast cancer, radiotherapy, chemotherapy, diabetes, stroke, chronic obstructive pulmonary disease, and heart failure were assessed at baseline	Age, census region, index year, Charlson index, number of drug classes used, statin use at baseline, PPI use at baseline, insurance produce, urban/rural residence, median household income in zip code, % in high school education in zip, % blacks in zip, % Hispanics in zip.
Relative risk taken from paper, or calculated from raw numbers	Paper	Paper	Paper
CVD outcome(s)	MI	Thromboembolic events	Stroke, MI

**Author**	Chen	Yang	Abdel-Qadir
Year	2014	2014	2016
Title	No increased venous thromboembolism risk in Asian breast cancer patients receiving adjuvant tamoxifen	Association of tamoxifen use and reduced cardiovascular events among asian females with breast cancer	The risk of myocardial infarction with aromatase inhibitors relative to tamoxifen in post-menopausal women with early stage breast cancer
Country	Taiwan	Taiwan	Canada
Study Type	Observational	Observational	Observational
Data source	Taiwan Cancer Registry Database	NHI Research Database Taiwan	Canadian administrative databases
Study Design	Cohort	Cohort	Cohort
Age	All patients	All patients	Post-menopausal
Inclusions	Diagnosed with stage I, II, or III breast cancer, according to the American Joint Committee on Cancer staging system (sixth version) criteria between January 1, 2004 and December 31, 2009; and received curative breast cancer surgery (lumpectomy or mastectomy) within 1 year after diagnosis	Patients who were newly diagnosed with breast cancer	Women with a first diagnosis of stage I-III breast cancer r between January 1, 2005 and December 31, 2010, who were dispensed a prescription for tamoxifen, or an aromatase inhibitor (i.e. anastrozole, letrozole or exemestane) within 1 year of cancer diagnosis, along with a second prescription dispensed within 1.5-times the number of days of the preceding prescription's supply
Exclusions	Women with history of other types of cancer or multiple primary invasive breast cancer; the presence of lymphoma (ICD-O-3 morphology code, 9590-9989), Kaposi's sarcoma (ICD-O-3 morphology code, 9140), and phyllodes tumor (IDC-O-3 morphology code, 9020) of the breast; received tamoxifen treatment prior to the operation date; and death within 28 days after the operation.	Patients with pre-existing cardiovascular disease, such as coronary artery disease, ischemic stroke, hemorrhagic stroke or peripheral artery disease were excluded	Women were excluded if they were treated with tamoxifen or AIs in the year preceding breast cancer diagnosis. Women were also excluded if they had substantial exposure to both tamoxifen and an aromatase inhibitor. This was defined as >10% of the days during which either tamoxifen or an aromatase inhibitor was prescribed. Accordingly, women were only included if they were exposed to one of the drug categories for 90% of the days during which a study drug was dispensed.
Intervention arm	At least one tamoxifen prescription after the index date (n=17874)	at least one tamoxifen prescription associated with the breast cancer diagnosis (n=2056)	Aromatase inhibitors at index date (n=7049). Patients were analysed based on the drug category they were predominantly exposed to.
Reference arm	No tamoxifen prescriptions after the index date (n=10155). No information about AI prescriptions.	No tamoxifen (n=1634). No information about AI prescriptions.	Tamoxifen at index date (n=1941)
Primary end point	Deep vein thrombosis/Pulmonary embolism. DVT estimates used in systematic review results	AMI, ischemic stroke, hemorrhagic stroke and total cardiovascular events	Myocardial infarction (hospitalisation)
Follow up time	Median follow up 48 months (range 0 -96)	Mean follow up 82.8 months	Mean follow up 39.9 months

Statistical methods (if applicable/available for CVD outcome)	Outcomes were compared using the Cox proportional hazard model for estimating hazard ratios and 95 % confidence intervals.	Survival analysis was assessed using Kaplan-Meier analysis, with the significance based on the log-rank test. The survival time was calculated from the date of enrollment to the development of AMI, ischemic stroke or hemorrhagic stroke. Multiple regression analysis was carried out using Cox proportional hazard regression analysis to evaluate the effect of tamoxifen use on determining the occurrence of AMI, stroke, or total cardiovascular events.	Time-to-event analyses were performed for MI, using tamoxifen as the reference treatment. Cumulative incidence function curves were used to estimate the cumulative incidence of MI over time after accounting for the competing risk of death. This allowed us to estimate the incidence of MI, given that some subjects will die before the occurrence of a cardiac event. IPTW using the propensity score was used to reduce the effects of measured confounding variables when estimating the effect of AIs versus tamoxifen. The PS model was estimated using a logistic regression model with receipt of AIs as the dependent variable and all covariates as the independent or explanatory variables. The variables that were chosen included markers of cardiovascular disease, cardiovascular risk factors, cancer severity, major non-cardiovascular co-morbidities, health care utilisation, factors that increase risk of adverse cardiac events with breast cancer (left-sided disease, chemotherapy, trastuzumab, radiation), as well as medications that could impact risk of cardiovascular disease. Truncated weights were used to minimise undue influence from atypical individuals with very high weights. The distribution of measured baseline covariates was compared between treatment groups in the sample weighted by the inverse probability of treatment using standardised differences. Variables were determined to be well balanced if the standardised difference was
Adjustments	Congestive heart failure, rheumatic disease, renal disease, diabetes mellitus, hypertension, Charlson comorbidity index. Patients had to have been diagnosed with the comorbidity within 1 year prior to the index date. Information was obtained from the NHI database, and all of the diagnoses were identified from either a single report in the inpatient medicinal claims file or from no less than two reports in the outpatient medicinal claims files	Diabetes mellitus, cardiac arrhythmia, hyperlipidemia, congestive heart failure, and chronic obstructive pulmonary disease, 365 days before the date of diagnosis of breast cancer. The diagnosis code of any comorbidity must have appeared at least twice and lasted longer than 30 days before officially being regarded as a comorbidity. Medications before enrollment were also reviewed within the database, which included angiotensin-converting enzyme inhibitors, β-adrenergic antagonists, calcium-channel blockers, diuretics, statins, antiplatelet agents (aspirin or clopidogrel) and thiazides.	Factors used in IPTW were: age, income quintile, rural residence, year of cohort entry, breast cancer side, chemotherapy, radiation, trastuzumab, CVD (other than MI), diabetes, dyslipidemia, hypertension, venous thromboembolism, fracture, renal disease, dialysis, prior malignancy, Charlson index, primary care visits in past year, specialist visits in past year, total physician visits in past year, medications dispensed in past year, ACE inhibitor, ARB, aspirin, thienopyridines, beta-blockers, calcium channel blockers, digoxin, aldosterone antagonists, diuretics, statins, oral hypoglycemics, insulin, vitamin K antagonists, low molecular weight heparin, nitrates, NSAIDs
Relative risk taken from paper, or calculated from raw numbers	Paper	Paper	Paper
CVD outcome(s)	Thromboembolic events	Stroke, MI	MI

**Author**	Haque	Rutqvist	McDonald
Year	2016	1993	1995
Title	Cardiovascular Disease After Aromatase Inhibitor Use	Cardiac and thromboembolic morbidity among postmenopausal women with early-stage breast cancer in a randomized trial of adjuvant tamoxifen. The Stockholm Breast Cancer Study Group	Cardiac and vascular morbidity in women receiving adjuvant tamoxifen for breast cancer in a randomised trial. The Scottish Cancer Trials Breast Group
Country	USA	Sweden	Scotland
Study Type	Observational	RCT	RCT
Data source	Kaiser Permanente Southern California		
Study Design	Cohort		
Age	Post-menopausal	Post-menopausal	<80 years old
Inclusions	Women with a first diagnosis of primary breast cancer between 1991 and 2010 and observed them through December 2011. For eligibility, women had to have pharmacy benefits, and have estrogen– or progesterone receptor–positive breast cancer.	Histologically verified invasive breast cancer, and no previous history of cancer	Early invasive breast cancer suitable for mastectomy
Exclusions	Prior CVD (cardiac ischemia (acute myocardial infarction and angina), stroke, heart failure and cardiomyopathy, and other events (dysrhythmia, valvular dysfunction, and pericarditis))	Inoperable local disease or distant metastasis at the time of primary diagnosis, other concurrent cancers, medical contraindications to the therapy, and operation which deviated from the protocol	T4, N2, N3 or Mi lesions, more than one palpable malignant lesion or bilateral breast cancer, In-situ carcinoma (including Paget's disease) without proof of underlying or associated invasive carcinoma, Patients who were or wished to become pregnant, Those unwilling to discontinue unrelated hormone therapy including the contraceptive pill, Previous malignant disease other than successfully treated squamous or basal cell carcinoma of skin, Previous systemic therapy for breast cancer, Any cause likely to compromise adequate review, premenopausal women with proven involvement of axillary lymph nodes who were enrolled in a trial comparing ovarian ablation with chemotherapy as adjuvant treatment
Intervention arm	Current aromatase inhibitors only (n=3807)	Tamoxifen (40mg) daily for 2 years (n=203)	20mg tamoxifen daily for 5 years (n=661)
Reference arm	Current tamoxifen only (n=4207)	No tamoxifen (n=219). Unlikely to have been prescribed AIs due to study period being before approval of AIs.	No tamoxifen (n=651). Unlikely to have been prescribed AIs due to study period being before approval of AIs.
Primary end point	CVD events (cardiac ischemia (acute myocardial infarction and angina), stroke, heart failure and cardiomyopathy, and other events (dysrhythmia, valvular dysfunction, and pericarditis)	Cardiac and thromboembolic morbidity	Adverse events, including CVD
Follow up time	72886 person-years of follow-up	Median follow up 60 months	5 years of tamoxifen, then could be randomized to receive more tamoxifen after this

Statistical methods (if applicable/available for CVD outcome)	Follow-up commenced on the breast cancer diagnosis date and ended on the date of one of the study end points (first CVD diagnosis date, death, termination of health plan membership, or study's end [December 31, 2011]), whichever occurred first. Hazard ratios (HRs) and 95% CIs were estimated using<br>Cox proportional hazards models with time-dependent medication use variables	All analyses were on the basis of "intention to treat." All patient data were analyzed according to the allocated treatment regardless of whether the patient actually received that treatment. No patient randomly assigned to treatment was excluded from analysis. Number of outcomes reported for adverse events.	Cox proportional hazards with censoring at date of systemic relapse, death, or at follow up to 31 December 1992.
Adjustments	Age at diagnosis, diagnosis year, breast cancer stage, race/ethnicity (from the SEER registry), geocoded median household income, body mass index, medical center, tumor characteristics, and primary cancer treatment (surgery, radiotherapy, and chemotherapy). Comorbidities, captured in the year before breast cancer diagnosis, included hypertension, diabetes mellitus, and the Charlson comorbidity index score. Data on pharmacy use related to CVD therapy and/or prevention were also extracted. These drug covariates were coded as binary (ever or never)		
Relative risk taken from paper, or calculated from raw numbers	Paper	Calculated	Calculated (HR calculated, but using incorrect reference group)
CVD outcome(s)	MI	Stroke, Heart failure	MI, Thromboembolic events

**Author**	Fisher	Fisher	Jakesz
Year	1999	2001	2005
Title	Tamoxifen in treatment of intra-ductal breast cancer: National Surgical Adjuvant Breast and Bowel Project B-24 randomised controlled trial	Tamoxifen and chemotherapy for axillary node-negative, estrogen receptor-negative breast cancer: findings from National Surgical Adjuvant Breast and Bowel Project B-23	Switching of postmenopausal women with endocrine-responsive early breast cancer to anastrozole after 2 years' adjuvant tamoxifen: combined results of ABCSG trial 8 and ARNO 95 trial
Country	USA	USA and Canada	Europe
Study Type	RCT	RCT	RCT
Data source			
Study Design			
Age	All patients	All patients	Post-menopausal
Inclusions	Women with DCIS were eligible for inclusion if their life expectancy was at least 10 years. Women with tumours that also consisted of DCIS and lobular carcinoma in situ (LCIS) were eligible. Women had to undergo lumpectomy.	primary operable, histologically node-negative, ER-negative breast cancer and a life expectancy of at least 10 years	Eligible patients were postmenopausal women aged 80 years or younger (ABCSG trial 8) or 75 years or younger (ARNO 95) with histologically verified, locally radically treated invasive or minimally invasive breast cancer without previous chemotherapy, hormone therapy, or radiotherapy, and absence of organ metastases. Women must have had 2yrs of tamoxifen (20mg) daily.
Exclusions	Women who had previously been diagnosed with cancer, except for those who had had in-situ carcinoma of the cervix or squamous-cell or basal-cell carcinoma of the skin, were not eligible.	No information given	indeterminate menopausal status (or menopausal status maintained by medication), presence of secondary malignant disease, tumour infiltration of skin or breast muscle (T4 tumours), and presence of other concomitant serious medical conditions—eg, those involving bone marrow function, the central nervous system, uncompensated cardiac insufficiency, or uncontrolled local or systemic infection.
Intervention arm	Radiation therapy followed by tamoxifen (10mg) twice daily for 5yrs (n=891)	CMF and tamoxifen (10 mg) twice a day (n=498)	Anastrozole (1mg) daily for remainder of 5 year endocrine treatment following 2 years of tamoxifen (n=1602). Follow up began after initial tamoxifen.
Reference arm	Radiation therapy followed by placebo (n=890). Unlikely to have been prescribed AIs due to study period being before approval of AIs.	CMF and placebo (n=499). Unlikely to have been prescribed AIs due to study period being before approval of AIs.	Tamoxifen (20mg) daily for remainder of 5yr endocrine treatment following 2 initial years of tamoxifen (n=1597). Follow up began after initial tamoxifen.
Primary end point	Disease free survival	Disease free survival	Disease free survival
Follow up time	Median follow up 74 months (range = 57-93 months)	Mean follow up 65 months (range 10 to 102 months)	Median follow-up 28 months (95% CI: 26–30)
Statistical methods (if applicable /available for CVD outcome)	Number of outcomes reported for adverse events.	Number of outcomes reported for adverse events.	Adverse events were only counted once per patient, and are described with absolute frequencies and proportions. Differences in the adverse event rates were estimated with exact odds ratios (OR) and corresponding 95% CIs. Exact ORs stratified by country were calculated for the five types of serious adverse events available for Austrian and German patients (myocardial infarct, embolism, thromboses, fractures, and endometrial cancer).
Adjustments			
Relative risk taken from paper, or	Calculated	Calculated	Paper

calculated from raw numbers			
CVD outcome(s)	Stroke, MI, Thromboembolic events	Thromboembolic events	Thromboembolic events, MI

**Author**	Goss	Boccardo	Coombes
Year	2005	2006	2007
Title	Randomized Trial of Letrozole Following Tamoxifen as Extended Adjuvant Therapy in Receptor-Positive Breast Cancer: Updated Findings from NCIC CTG MA.17	Switching to anastrozole versus continued tamoxifen treatment of early breast cancer. Updated results of the Italian tamoxifen anastrozole (ITA) trial	Survival and safety of exemestane versus tamoxifen after 2-3 years' tamoxifen treatment (Intergroup Exemestane Study): a randomised controlled trial
Country	Canada	Italy	UK
Study Type	RCT	RCT	RCT
Data source			
Study Design			
Age	Post-menopausal	Post-menopausal	All patients
Inclusions	Previous adjuvant tamoxifen therapy lasting 4.5 – 6 years; histologically confirmed primary breast cancer; a tumor that was positive for estrogen receptor, progesterone receptor, or both (defined by a level of 10 fmol/mg protein or a positive result on immunohistochemical analysis of ER or PR); discontinuation of tamoxifen therapy less than 3 months before enrollment; an Eastern Cooperative Oncology Group performance status of 0, 1, or 2 (scored on a scale of 0 to 4, with lower scores indicating better function); a life expectancy of more than 5 years; and postmenopausal status. Women were defined as being postmenopausal if they were at least 50 years of age at the start of adjuvant tamoxifen therapy, were younger than 50 years at the start of tamoxifen therapy but postmenopausal at the initiation of tamoxifen therapy, were younger than 50 years at the start of tamoxifen therapy but had undergone bilateral oophorectomy, were premenopausal and younger than 50 years of age at the start of tamoxifen therapy but became amenorrheic during chemotherapy or treatment with tamoxifen, or were any age but had postmenopausal levels of luteinizing hormone or follicle-stimulating hormone prior to study enrollment. Women with unknown hormone receptor status were eligible, provided an effort was made to determine the receptor status of the primary tumor.	Histologically confirmed primary breast cancer, tumor estrogen receptor positivity , positive axillary nodes, and no evidence of recurrent or metastatic disease, who were receiving adjuvant treatment with tamoxifen for the last 2–3 years	Patients were eligible if they had histologically confirmed, completely resected unilateral invasive breast carcinoma that was positive for estrogen receptors or that was of unknown receptor status. Patients were postmenopausal and had received adjuvant tamoxifen therapy for at least two years but not more than three years and one month. Patients were required to have adequate hematologic, renal, and liver function at the time of randomization. b
Exclusions	No information given	Patients with a history or presence of any other cancer (except adequately treated skin cancer or carcinoma-in-situ of the cervix) and patients with any condition that may jeopardize their compliance to treatment or follow-up	The presence of a tumor with known negative estrogen-receptor status; evidence of local relapse or a distant metastasis since the time of diagnosis; a clinically significant skeletal, cardiac, or endocrine disorder; and the use of hormone-replacement therapy within four weeks before randomization. Patients were also excluded if they had clinical evidence of severe osteoporosis or a history of a previous neoplasm other

			than carcinoma in situ of the cervix or basal-cell skin carcinoma or if they were taking concomitant anticoagulant agents, a selective estrogen-receptor modulator other than tamoxifen, or any other form of hormonal therapy.
Intervention arm	Letrozole (2.5mg) daily for 5 years, following previous adjuvant tamoxifen for 4.5-6 years. Follow up began after initial tamoxifen.	1mg of anastrozole daily for remainder of 5yr endocrine treatment following 2-3yrs of tamoxifen (n=223). Follow up began after initial tamoxifen.	Exemestane (25mg) daily for remainder of 5yr endocrine treatment following 2-3 years of tamoxifen (n=2320). Follow up began after initial tamoxifen.
Reference arm	Placebo daily for 5 years, following previous adjuvant tamoxifen for 4.5-6 years. Follow up began after initial tamoxifen.	20mg of tamoxifen daily for the remainder of their 5yr endocrine treatment following 2-3 initial yrs of tamoxifen (n=225). Follow up began after initial tamoxifen.	Tamoxifen (20 or 30mg) daily for the remainder of their of 5yr endocrine treatment following 2-3 initial years of tamoxifen (n=2338). Follow up began after initial tamoxifen.
Primary end point	Disease free survival	Disease free survival	Disease free survival
Follow up time	Median follow-up 30 months (range 1.5 - 61.4 months)	Median follow up 64 months (range = 12-92 months)	Median follow up 55.7 months (range = 0-89.7 months)
Statistical methods (if available for CVD outcome)	Number of outcomes reported for adverse events.	Number of outcomes reported for adverse events.	Number of outcomes reported for adverse events.
Adjustments			
Relative risk taken from paper, or calculated from raw numbers	Calculated	Calculated	Calculated
CVD outcome(s)	Angina, Stoke, MI, Thromboembolic events	Thromboembolic events	MI, PVD

**Author**	Kaufmann	Forbes	Abo-Touk
Year	2007	2008	2010
Title	Improved survival in postmenopausal women with early breast cancer after anastrozole initiated after treatment with tamoxifen compared with continued tamoxifen: The ARNO 95 study	Effect of anastrozole and tamoxifen as adjuvant treatment for early-stage breast cancer: 100-month analysis of the ATAC trial	Switching to Letrozole Versus Continued Tamoxifen Therapy in Treatment of Postmenopausal Women with Early Breast Cancer
Country	Germany	International	Egypt
Study Type	RCT	RCT	RCT
Data source			
Study Design			
Age	Post-menopausal	Post-menopausal	Post-menopausal
Inclusions	Women with histologically verified, grade 1 to 3 invasive breast cancer (pT1-3, node negative, or up to nine tumour-infiltrated lymph nodes [pN0-2] and no distant metastases), who had undergone primary surgery (with or without radiotherapy) and had received 2 years of continuous adjuvant tamoxifen (20 or 30 mg/d) with disease recurrence	women with histologically proven operable invasive breast cancer who had completed primary surgery and chemotherapy (where given), and were candidates to receive hormonal adjuvant therapy. Patients with negative or unknown hormone-receptor status were included because hormone-receptor-negative patients were thought to derive benefit from adjuvant therapy with a hormonal agent.	Histologically confirmed operable invasive early breast carcinoma with positive estrogen, or progesterone receptors, or both. Primary surgery was modified radical mastectomy or breast conserving surgery with axillary lymph-node dissection with resulting clear margins. There was no evidence of metastatic or recurrent disease; previous or concurrent cancer. Adequate hematologic, renal and hepatic functions were required
Exclusions	No information given	Patients were ineligible if there was any clinical evidence of metastatic disease; if chemotherapy was started more than 8 weeks after surgery or completed more than 8 weeks before starting randomised treatment (neo-adjuvant chemotherapy was not allowed) or, in patients not receiving chemotherapy, if primary surgery was completed more than 8 weeks before starting randomised treatment; or if they had received hormonal therapy for breast-cancer prevention or for adjuvant treatment of breast cancer (except if tamoxifen treatment was started before surgery and received for less than 29 days, or if hormonal therapy was received before surgery in the context of a formal trial previously approved by the Steering Committee). Patients were not eligible if they were unwilling to stop any hormonal drug including HRT; if they had a previous history of invasive malignant disease (breast cancer at any time, other malignant disorders within the past 10 years excluding squamous or basal-cell carcinoma of the skin or carcinoma in situ of the cervix, adequately cone biopsied); or if the patient had any severe concomitant disease which would place the patient at unusual risk or confound the results of the trial. Patients were included o	No information given
Intervention arm	Tamoxifen (20 or 30mg) daily for 2 years followed by anastrozole (1mg) daily for	Anastolzole only after surgery (n=3125)	Tamoxifen (20mg) daily for 2 years followed by letrozole (2.5mg) daily for another 3

	another 3 years (n=445). Follow up began after initial tamoxifen.		years. Follow up began after initial tamoxifen.
Reference arm	Tamoxifen (20 or 30mg) daily for the remainder of their 5yr endocrine treatment following 2 years of initial tamoxifen (n=452). Follow up began after initial tamoxifen.	Tamoxifen only after surgery (n=3116)	Tamoxifen (20mg) daily for 3 years following 2 years initial tamoxifen. Follow up began after initial tamoxifen.
Primary end point	Disease free survival	Disease free survival	Disease free survival
Follow up time	Median follow up 30.1 months	Median follow up 100 months (range 0–126)	Median follow up 41 months (range 15 to 62 months)
Statistical methods (if available for CVD outcome)	Number of outcomes reported for adverse events.	Side-effects were summarised according to the hormone treatment first received. Except for other cancers, side-effect events were accrued up to 14 days after stopping treatment. Information on new primary cancers was collected during and after trial treatment (before and after recurrence), but only summarised up to the point of recurrence. The comparisons of pre-specified adverse events were based on a simple comparison of proportions, and Fisher's exact two-sided p values were used when necessary.	Number of outcomes reported for adverse events.
Adjustments			
Relative risk taken from paper, or calculated from raw numbers	Calculated	Calculated	Calculated
CVD outcome(s)	Stroke	Stroke, MI, Thromboembolic events	Stroke

**Author**	Colleoni	van de Velde	Bliss
Year	2011	2011	2012
Title	Analyses Adjusting for Selective Crossover Show Improved Overall Survival With Adjuvant Letrozole Compared With Tamoxifen in the BIG 1-98 Study	Adjuvant tamoxifen and exemestane in early breast cancer (TEAM): a randomised phase 3 trial	Disease-related outcomes with long-term follow-up: an updated analysis of the intergroup exemestane study
Country	International	Europe	International
Study Type	RCT	RCT	RCT
Data source			
Study Design			
Age	Post-menopausal	Post-menopausal	Post-menopausal
Inclusions	Patients were eligible for the study if they had tumors that were positive for estrogen receptors, progesterone receptors, or both. Primary surgery with resulting clear margins and adequate hematologic, renal, and hepatic function were required.	Histologically confirmed breast adenocarcinoma and locally assessed oestrogen-receptor-positive or progesterone-receptor-positive disease who had completed local treatment administered with curative intent. Other eligibility criteria were invasive tumours of all sizes, with or without involvement of the lymph nodes (N0 to N3) and no evidence of metastatic disease.	ER–positive/ER-unknown primary invasive breast cancer who remained disease-free and on treatment after 2 to 3 years of tamoxifen, with adequate hematologic, renal, and liver function at the time of randomization
Exclusions	Evidence of metastatic disease; previous or concurrent cancer other than adequately treated noninvasive breast or cervical cancer or basal-cell or squamous-cell carcinoma of the skin within 5 years before randomization; receipt of adjuvant antiestrogen therapy for the primary breast cancer for at least 1 month; and treatment with systemic investigational drugs within 30 days before randomization or topical investigational drugs within 7 days before randomization.	Patients were excluded if they had substantial cardiac disease, other malignant diseases, or illnesses interfering with participation in the study. Further details have been previously reported.	Presence of a tumor with known negative estrogen-receptor status; evidence of local relapse or a distant metastasis since the time of diagnosis; a clinically significant skeletal, cardiac, or endocrine disorder; and the use of hormone-replacement therapy within four weeks before randomization. Patients were also excluded if they had clinical evidence of severe osteoporosis or a history of a previous neoplasm other than carcinoma in situ of the cervix or basal-cell skin carcinoma or if they were taking concomitant anticoagulant agents, a selective estrogen-receptor modulator other than tamoxifen, or any other form of hormonal therapy. The protocol required adequate treatment of primary disease, including postoperative radiotherapy in patients who had been treated with breast-preserving surgery. Neo-adjuvant chemotherapy was permitted according to a consistent policy within each center. Patients were required to have started chemotherapy within three months after diagnosis and to have begun receiving tamoxifen and radiotherapy within three months after the completion of chemotherapy.
Intervention arm	1) Letrozole (2.5mg) daily for 2 years followed by tamoxifen (25mg) daily for 3 years (n=1540), 2) Tamoxifen (25mg) daily for 2 years followed by letrozole (2.5mg) daily for 3 years (n=1548). Follow up began after initial 2 year treatment..	Tamoxifen (20 mg) daily for 2-3yrs followed by Exemestane (25 mg) daily for the remainder of the 5yrs (n=4868). Follow up for the whole period.	25mg of exemestane daily for remainder of 5yr endocrine treatment following 2-3 years of tamoxifen (n=2105). Follow up began after initial tamoxifen.

Reference arm	1) Only tamoxifen (25mg) daily for 5 years (n=1548), 2) Only letrozole (2.5mg) daily for 5 years (n=1546). Follow up began after initial 2 year treatment.	Exemestane (25 mg) daily for 5yrs (n=4898). Follow up for the whole period.	20 or 30mg of tamoxifen daily for the remainder of their of 5yr endocrine treatment following 2-3 initial years of tamoxifen (n=2036). Follow up began after initial tamoxifen.
Primary end point	Disease free survival	Disease free survival	Disease free survival
Follow up time	Media follow up 74 months	Median follow-up 5·1 years	Median follow up 91 months (IQR=83-99.2 months)
Statistical methods (if available for CVD outcome)	Selective crossover then number of outcomes reported for adverse events.	Number of outcomes reported for adverse events.	Kaplan-Meier plots, log-rank tests, and Cox proportional hazards models were used.
Adjustments			
Relative risk taken from paper, or calculated from raw numbers	Calculated	Calculated	Paper
CVD outcome(s)	Stroke, Heart failure, Thromboembolic events	Arrhythmia, Heart failure, MI, Thromboembolic events	Angina, Thromboembolic events

**Author**	Pagani	Hernandez
Year	2014	2008
Title	Adjuvant Exemestane with Ovarian Suppression in Premenopausal Breast Cancer	Tamoxifen Treatment in Danish Breast Cancer Patients and 5-Year Risk of Arterial Atherosclerotic Events: A Null Association
Country	International	Denmark
Study Type	RCT	Observational
Data source		Danish Registries
Study Design		Cohort
Age	Pre-menopausal	45-69 years old
Inclusions	Histologically proven operable breast cancer confined to the breast and ipsilateral axilla, with the exception of internal-mammary-node involvement detected by means of sentinel-node biopsy, and tumor that expressed estrogen or progesterone receptors in at least 10% of the cells, as assessed with the use of immunohistochemical testing. Patients with synchronous bilateral hormone-receptor–positive breast cancer were eligible. Patients had undergone either a total mastectomy with subsequent optional radiotherapy or breast-conserving surgery with subsequent radiotherapy. Either axillary dissection or a negative sentinel-node biopsy was required. Macrometastasis in a sentinel node required axillary dissection or irradiation.	Women eligible for the study were diagnosed with International Union Against Cancer stage I or stage II estrogen receptor-positive breast cancer between 1990 and 2004 at ages 45 to 69 years, as reported to the Danish Breast Cancer Cooperative Group (DBCG) clinical database
Exclusions		Women with no existing cardiovascular disease (defined using ICD-8 and ICD-10 codes) as of the date of breast cancer surgery
Intervention arm	In the TEXT study, 5 yrs of exemestane (25mg daily) plus triptorelin. In the SOFT study 5 yrs of exemestane plus ovarian supression.	Any tamoxifen during follow up (n=8232)
Reference arm	In the TEXT study 5 yrs of tamoxifen (20mg daily) plus triptorelin. In the SOFT study 5 yrs of tamoxifen plus ovarian supression.	Unexposed to tamoxifen. Unlikely to have been prescribed Ais due to study period being before approval of AIs (n=8057)
Primary end point	Disease free survival	Angina, MI, HF, stroke
Follow up time	Median follow up of 68 months	Not reported
Statistical methods (if available for CVD outcome)	Number of outcomes reported for adverse events.	Follow-up was initiated 3 months after the surgery date. Follow-up ended on December 31, 2005. Risks of events were analyzed individually by year for the first 5 years of follow-up, and then cumulatively for Years 1 to 5. RRs and 95% confidence intervals were calculated as estimates of the association between tamoxifen therapy and incident CVD events. Cox proportional hazards models were used to estimate crude HRs and adjusted HRs controlling for confounding, for years 1 to 5 individually, and for Years 5 to 10 taken together. The

		proportional hazards assumption was tested by adding a covariate to the model to represent the interaction between exposure and the log of survival time
Adjustments		Age group, diabetes, renal disease, hypertension, chronic obstructive pulmonary disease, radiation therapy, and chemotherapy
Relative risk taken from paper, or calculated from raw numbers	Calculated	Paper
CVD outcome(s)	Stroke, MI, Thromboembolic events	Angina, stroke, MI, heart failure

Appendix 6 – Bias assessment of RCTs

Study	Random sequence generation	Allocation concealment	Blinding	Incomplete outcome data	Selective reporting	Other sources of bias
Bliss 2012	1 - permuted blocks	0	1 - double blinded	1 - All patients included	1 - CVD events were coded according to criteria specified by an independent cardiologist	1 - No other risks of bias
Boccardo 2006	0	0	0	1 - All patients included	0	1 - No other risks of bias
Coombes 2007	1 - permuted blocks	0	1 - double blinded	1 - 95% had full follow up	0	1 - No other risks of bias
Fisher 1999	0	0	0	1 - All patients included	0	1 - No other risks of bias
Fisher 2001	1- biased coin	0	0	1 - 98% had full follow up	0	1 - No other risks of bias
Forbes 2008	1 - randomisation by computer	1 - central allocation	0	0	0	1 - No other risks of bias
Jakesz 2005	1 - randomisation by computer	1 - central allocation	2 - open label trials	0	0	1 - No other risks of bias
Kaufmann 2007	1 - randomisation by computer	1 - central allocation	0	1 - 6 patients discontinued	0	1 - No other risks of bias
McDonald 1995	0	0	0	0	0	1 - No other risks of bias
Colleoni 2011	1 - permuted blocks	0	1 - double blinded	1 - All patients included	0	1 - No other risks of bias
Rutqvist 1993	0	0	0	1 - Outcome data taken from registries	0	1 - No other risks of bias
van de Velde 2001	1 - randomisation by computer	1 - Only statistician and steering committee had access to unmasked data	2 - open label trial	0	0	1 - No other risks of bias
Abo-Touk 2010	1 - simple randomisation method	0	0	1 - All patients included	0	1 - No other risks of bias
Goss 2005	1 - Minimisation method	0	1 - double blinded	1 - All patients included	0	1 - No other risks of bias
Pagani 2014	1 - permuted blacks	0	2 - open label trials	1 - All patients included	0	1 - No other risks of bias

Bias assessment categories
0 - No information given
1 - Low risk of bias
2 - High risk of bias

Appendix 7 – Bias assessment of observational studies

Study	Exposure definition	Outcome/case definition	Control selection	Confounding	Missing Data	Censoring
Abdel-Qadir 2016	2 - Only included women who were exposed to either AI or Tam for >90% of days dispensed	2 - Used hospital records, but not clear what method used to define outcome	N/A	1 - Adjustment for a wide range of confounders using IPTW	0 - No information given	1 - Only censored at end of study
Chen 2014	2 - Patients only needed one tamoxifen prescription to be defined as exposed	1 - Use hospital records and outlines ICD-9 codes used	N/A	2 - No adjustment for CVD related treatment, cancer severity, or other cancer treatments	0 - No information given	1 - Only censored at end of study or death
Haque 2016	2 - Patients only needed one tamoxifen prescription to be defined as exposed	1 - Identified by medical records and validated by clinician	N/A	1 - Adjustment for wide range of covariates and used IPTW	1 - Missing data on BMI, but sensitivity analyses performed to assess the impact of this	1 - Censored at death or termination of health plan membership
Hernandez 2008	0 - Not enough information given	1 - Outcome defined by ICD 8 and 10 codes	N/A	1 - Adjustment for wide range of covariates	0 - No information given	1 - Only censored at outcome or end of follow-up
Hernandez 2009	0 - Not enough information given	1 - Outcome defined by ICD 8 and 10 codes	N/A	1 - Adjustment for wide range of covariates	2 - A lot of missing BMI data and no explanation of how it is dealt with	1 - Only censored at outcome or end of follow-up
Ligibel 2012	2 - Ascertained through pharmacy data, but no information on how exposure begins	1 - Outcome identified through hospital records	N/A	2 - No adjustment for cancer severity or other cancer related treatments	0 - No information given	1 - Only censored at outcome or end of follow-up
Yang 2014	2 -Not clear when patient defined as exposed, and all breast cancer patients could be unexposed, even with ER- BC	1 - based on ICD-9 from medical records	N/A	2 - No adjustment for cancer severity or other cancer treatment	0 - No information given	0 - No information given
Bradbury 2005	2 - Patients are taken from a study population that include bladder, colorectal, and non-melanoma skin cancer patients, who would not be prescribed tamoxifen	2 - Used GP records, but no indication of terms used to define the case	1 - Three controls matched on date of IHD diagnosis, age, and study entry date	2 - No adjustment for CVD related treatment, cancer severity, or other cancer treatments	1 - Minimal missing data	N/A
Geiger 2004	1 - Exposure abstracted from medical records for cases and controls	1 - Hospital records used	1 -Two controls matched on age and members of the same health maintenance organisation during their at-risk period	2 - All risk factors adjusted for, but breast cancer therapies, smoking and some medical therapies through patient recall	2 - Missing category fitted to deal with missing data	N/A
Geiger 2005	1 - Exposure abstracted from medical records for cases and controls	1 - Hospital records used	1 -Two controls matched on age and members of the same health maintenance organisation during their at-risk period	2 - All risk factors adjusted for, but breast cancer therapies, smoking and some medical therapies through patient recall	2 - Missing category fitted to deal with missing data	N/A
Meier 1998	1 - Ascertained through computerised medical records	1 - Based on hospital records	1 - Cancer free controls from GPRD population	2 - No adjustment for cancer severity or other cancer treatment	2 - Missing data category fitted	N/A

Bias assessment categories
0 - No information given
1 - Low risk of bias
2 - High risk of bias

Appendix 8 - Meta-analysis of observational studies examining the risk of heart failure in tamoxifen users compared to non-users

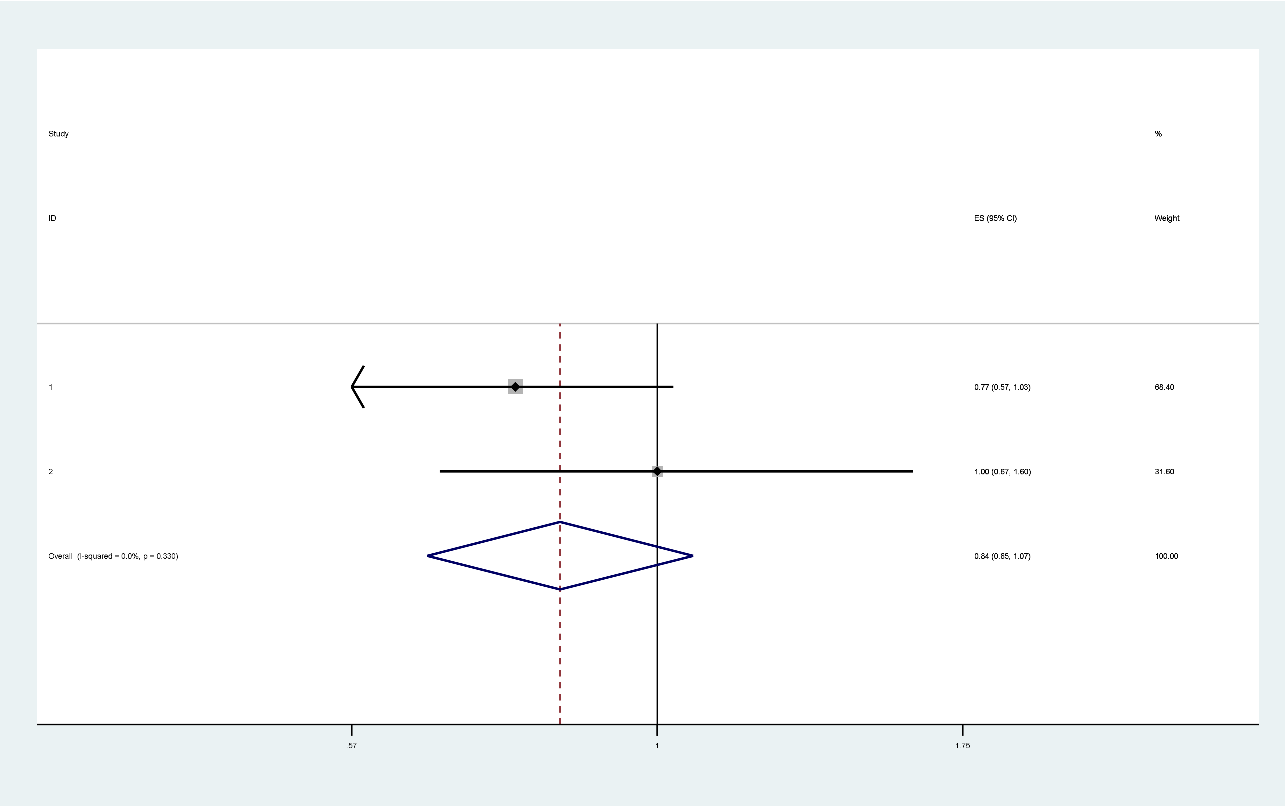

Appendix 9 - Meta-analysis of RCTs examining the risk of thromboembolic events in AI users compared to tamoxifen

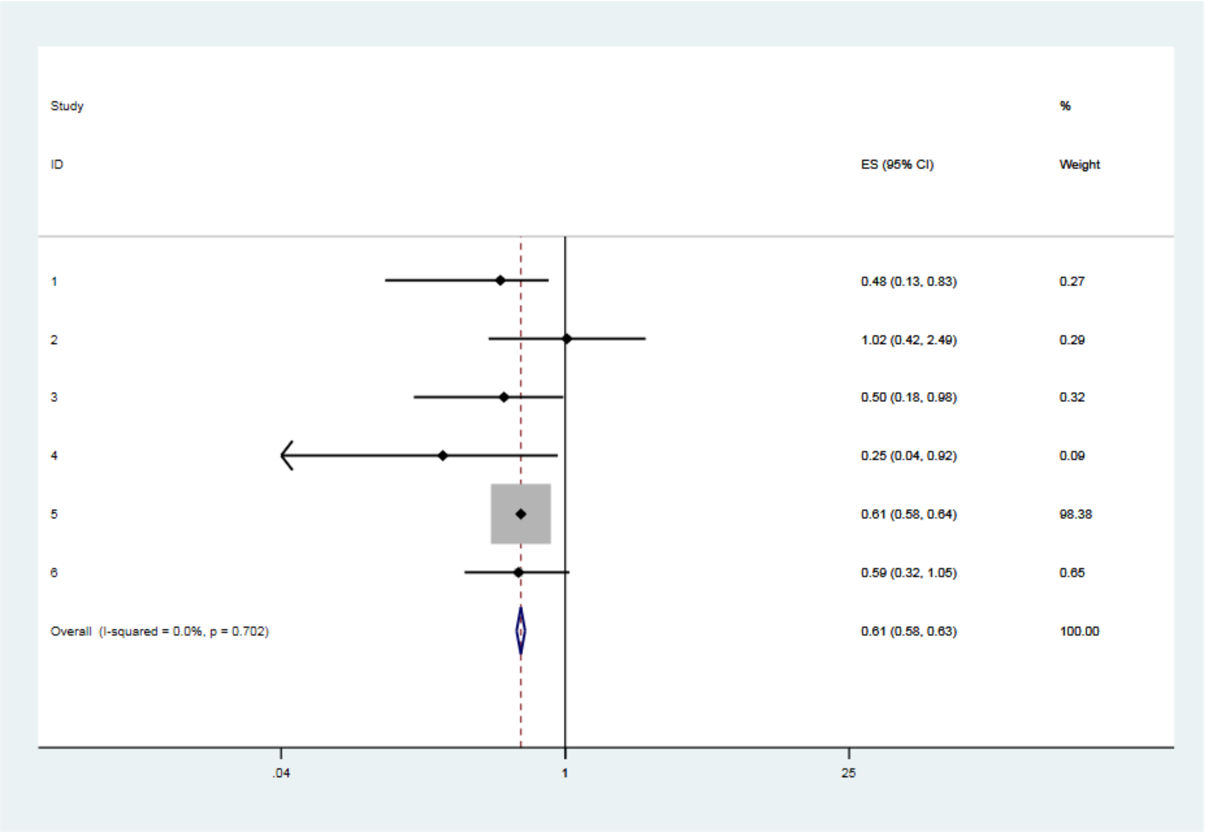

Supplement: Supplementary file 1 — Supplementary appendices [file mata044809.ww1.pdf]
